# Kynurenine 3-monooxygenase is a critical regulator of renal ischemia–reperfusion injury

**DOI:** 10.1038/s12276-019-0210-x

**Published:** 2019-02-13

**Authors:** Xiaozhong Zheng, Ailiang Zhang, Margaret Binnie, Kris McGuire, Scott P. Webster, Jeremy Hughes, Sarah E. M. Howie, Damian J. Mole

**Affiliations:** 1 Centre for Inflammation Research, University of Edinburgh, 47 Little France Crescent, Edinburgh, EH16 4TJ UK; 2 Centre for Cardiovascular Science, University of Edinburgh, 47 Little France Crescent, Edinburgh, EH16 4TJ UK

**Keywords:** Translational research, Experimental models of disease

## Abstract

Acute kidney injury (AKI) following ischemia–reperfusion injury (IRI) has a high mortality and lacks specific therapies. Here, we report that mice lacking kynurenine 3-monooxygenase (KMO) activity (*Kmo*^null^ mice) are protected against AKI after renal IRI. We show that KMO is highly expressed in the kidney and exerts major metabolic control over the biologically active kynurenine metabolites 3-hydroxykynurenine, kynurenic acid, and downstream metabolites. In experimental AKI induced by kidney IRI, *Kmo*^null^ mice had preserved renal function, reduced renal tubular cell injury, and fewer infiltrating neutrophils compared with wild-type (*Kmo*^wt^) control mice. Together, these data confirm that flux through KMO contributes to AKI after IRI, and supports the rationale for KMO inhibition as a therapeutic strategy to protect against AKI during critical illness.

## Kidney disease: drug target for acute injury following reperfusion

Inhibition of a metabolic enzyme linked to inflammation could be a novel treatment approach for sudden kidney failure following a “reperfusion” injury caused by blood flow returning to the organ after a period of insufficient blood supply. Damian Mole and colleagues from the University of Edinburgh, UK, temporarily blocked blood vessels leading to the kidneys of mice to induce organ damage. Mice that lacked a working copy of kynurenine 3-monooxygenase (*KMO*), a gene that encodes an enzyme involved in metabolizing an essential amino acid linked to immune activation, were protected from injury. These *KMO*-mutant mice experienced less damage to the kidney’s tubular cells and had fewer pro-inflammatory cells than genetically normal animals. The findings support the idea that blocking KMO and its associated metabolic pathway could help mitigate kidney damage following reperfusion injury in humans.

## Introduction

**Fig. 1:**
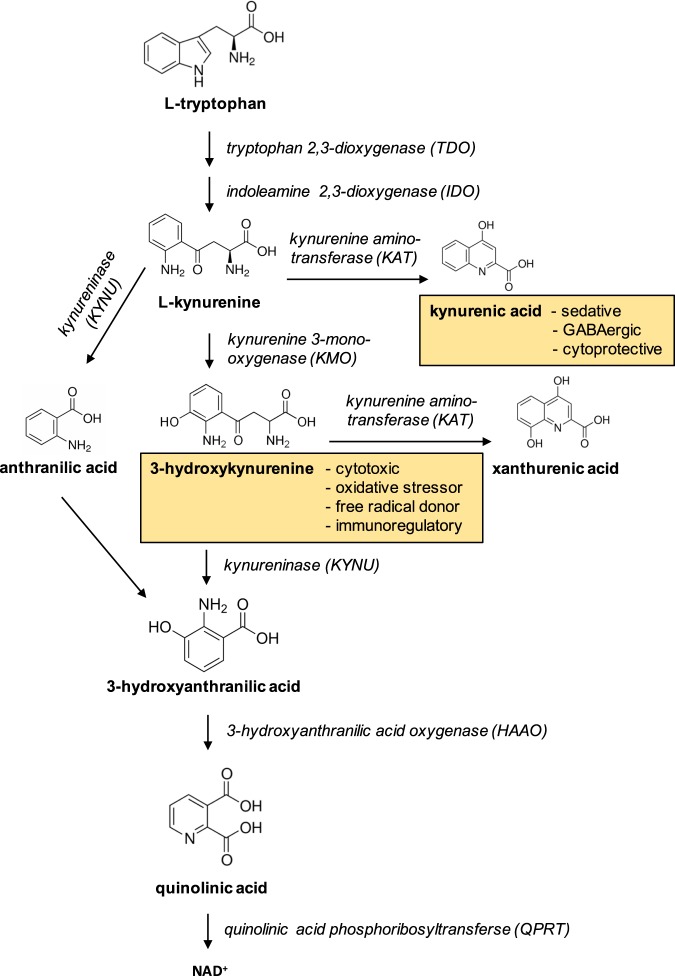
Overview of the kynurenine pathway of tryptophan metabolism. 3-Hydroxykynurenine, the product of the gate-keeper enzyme kynurenine 3-monooxygenase (KMO), and kynurenic acid, one of the other branch metabolite are highlighted

In eukaryotes, the metabolic fate of the essential amino-acid tryptophan is conversion via the kynurenine pathway into a range of metabolites that includes kynurenic acid, 3-hydroxykynurenine, and quinolinic acid. Enzymes involved in the metabolism of tryptophan along the kynurenine pathway are located throughout the body and brain, and are most abundant in the liver and kidney. The conversion of tryptophan to *N*-formylkynurenine (KYN) is catalyzed by tryptophan 2,3-dioxygenase (TDO) and indoleamine 2,3-dioxygenases (IDOs). The kynurenine pathway diverges at kynurenine into two distinct branches that are regulated by kynurenine aminotransferases (KATs) and kynurenine 3-monooxygenase (KMO), respectively (Fig. 1). KMO is the only route of 3-hydroxykynurenine production known to occur in humans. KMO localizes to the outer membrane of mitochondria, and is highly expressed in peripheral tissues, including liver and kidney^1^. KMO expression in mouse kidney is localized to the proximal tubule epithelial cells when measured by single-cell transcriptomics^2^ and KMO protein in humans is also localized to kidney tubule epithelial cells using immunohistochemistry^3^. 3-Hydroxykynurenine is injurious to several cell types^4^, causing tissue injury via oxidative stress, pathological cross-linking of proteins^5^, and inducing apoptotic cell death^6,7^. Kynurenine may also be metabolized to kynurenic acid by KATs and to anthranilic acid by kynureninase. Kynurenic acid is sedative^8^ and has been shown to be protective against cell injury in certain inflammatory situations^9,10^.

Renal ischemia–reperfusion injury (IRI) is a leading cause of acute kidney injury (AKI). AKI, as a component of multiple organ dysfunction syndrome (MODS), has a high mortality and lacks specific therapies. Renal IRI may occur as a result of primary decrease in blood flow, followed by reperfusion, for example, during abdominal aortic aneurysm repair or renal transplantation, or secondary to, and as part of a systemic inflammatory response, for example, during sepsis or severe acute pancreatitis (AP)^11^. The exact mechanisms that drive AKI in systemic inflammation and MODS are not well understood, but are likely to stem from a combination of hypoperfusion–reperfusion (IRI) and metabolic toxicity. In the context of secondary AKI, in experimental rodent models of AP-MODS, in which AKI is an important contributor, pharmacological inhibition of KMO, and separately, transcriptional blockade of the *Kmo* gene, reduces 3-hydroxykynurenine formation and protects against AKI^12–15^. However, whether *Kmo* gene blockade protects against primary IRI-induced AKI is not known. Therefore, to further elucidate the role of renal KMO in protecting against experimental AKI, we tested whether mice lacking functional KMO are protected from experimental AKI induced by the direct insult of renal IRI.

## Materials and methods

### Ethical considerations

All experiments were performed after Research Ethics Committee and Veterinary review at The University of Edinburgh, and were conducted according to the United Kingdom Use of Animals (Scientific Procedures) Act 1986, under license PPL60/4250.

### Animals

Embryonic stem cells on a C57BL/6N background were engineered to lack KMO activity by insertion of a polyA transcription “stop” motif before exon 5 of the *Kmo* gene (*Kmo*^*tm1a(KOMP)Wtsi*^) by the International Knockout Mouse Project (iKOMP). These mice were backcrossed with C57BL/6J mice to generate mice lacking KMO activity, hereafter referred to as *Kmo*^null^ mice as previously described^12^. Control mice (*Kmo*^*tm1c(KOMP)Wtsi/flox(ex5)*^), with normal *Kmo* gene transcription and KMO activity with *loxP*-flanked exon 5 of *Kmo* but no *cre*-recombinase expression and therefore a wild-type phenotype, hereafter referred to as *Kmo*^wt^ mice, were generated from the same founders as the *Kmo*^null^ strain. Genotyping of all mice was performed by polymerase chain reaction (PCR) using primers and a protocol published previously^12^. Male mice only were used. Mice were 10–15 weeks old and were housed under specific pathogen-free conditions in the Biomedical Research Resources Facility of the University of Edinburgh.

### Experimental IRI

Experimental kidney IRI was induced as described previously^16^. Briefly, mice were given a general anesthetic using intraperitoneal ketamine and metomidate according to local dosage guidelines. Under aseptic conditions, a midline laparotomy and right nephrectomy were performed. The left renal pedicle was identified and occluded by atraumatic clamp for 22 min. The duration of clamping was determined by our previous experience with this model using the same background mouse strain. Body temperature was maintained at 35 °C using a homeostatically controlled blanket (Harvard Apparatus, Boston, MA). After reperfusion, the abdominal wall was sutured closed with 5–0 polypropylene and the skin closed with metal clips. Anesthesia was reversed with Antisedan. Fluid resuscitation with 1 mL of sterile 0.9% NaCl was administered subcutaneously to the scruff after surgery. Sham-operated mice underwent general anesthesia, laparotomy, and unilateral nephrectomy, but no clamping of the left renal pedicle. The animals were singly housed and maintained in a 28 °C warm box overnight to recover from surgery. After 24 h, mice were humanely killed under terminal general anesthesia. Blood was collected into tubes containing EDTA (BD Biosciences). Urine was collected into sterile Eppendorf tubes. Whole kidneys were cut longitudinally and either snap frozen in liquid nitrogen for subsequent mRNA extraction or fixed in 10% neutral buffered formalin before embedding in paraffin for histological analysis.

### Assessment of renal function

Plasma samples were prepared from whole blood by centrifugation at 1000 × *g* for 5 min followed by aliquoting of the supernatant, freezing in dry ice and storage at −80 °C until analysis without freeze-thaw. Amylase, albumin, alanine aminotransferase, glucose, urea, and creatinine were analyzed by using commercial kits (Product codes are 17632H, 17600H, 17234H, 17630H, 17629H, and 17654H, respectively. Alpha Laboratories Ltd, Eastleigh, UK) adapted for use on a Cobas Fara centrifugal analyzer (Roche Diagnostics Ltd, Welwyn Garden City, UK). Specifically, creatinine was determined using the creatininase/creatinase-specific enzymatic method. Urinary albumin excretion was expressed as albumin/creatinine ratio (ACR).

### Histology and digital image analysis

Histological sections (4 μm) of formalin-fixed paraffin-embedded kidney tissue were de-waxed and taken through a decreasing series of graded alcohols to water. Hematoxylin and eosin (H&E) staining was performed according to standard protocols. The H&E-stained sections were scored in a blinded fashion for assessment of tubular necrosis in the outer medulla^17^. Ten representative random fields at a magnification of × 200 per section for each sample were examined. The percentage of tubules in the corticomedullary junction that displayed cellular necrosis and a loss of brush border were counted.

To assess the extent of apoptotic cell death induced by IRI, we performed terminal deoxynucleotidyl transferase dUTP-mediated nick-end labeling (TUNEL) staining on paraffin-embedded kidney tissue sections using a commercially available kit (DeadEnd fluorometric TUNEL system; Promega, Madison, WI, USA). Briefly, formalin-fixed sections of 4 μm thickness were deparaffinized, hydrated, and incubated with 20 μg/mL proteinase K to strip proteins from the nuclei. Fragmented DNA was then identified by the incorporation of fluorescein-12-dUTP during an incubation step with terminal deoxynucleotidyl transferase at 37 °C for 1 h. Sections were stained by immersing the slides in 40 mL of propidium iodide solution freshly diluted to 1 μg/mL in phosphate buffered saline for 15 min. Microscopic images were acquired at × 10 magnification by using a Leica DC350F digital camera system equipped with Nikon Eclipse E800 Fluorescence microscope and Image-Pro Plus image analysis software (Media Cybernetics). Apoptotic cells (TUNEL-positive cells) were quantitatively assessed at × 100 magnification for 13 fields of tubular areas in a blinded manner using ImageJ as previously described^12^.

We also performed cleaved caspase-3 immunohistochemistry from paraffin sections to detect renal apoptosis. Renal macrophages were identified by immunostaining for the tissue macrophage marker F4/80. Myeloperoxidase (MPO)-positive cells were quantified in post-ischemic kidney sections as an index of neutrophil infiltration. The following primary antibodies were used: cleaved caspase-3 (ASP175) antibody with cat no. 9661 (Cell Signaling, Danvers, MA, USA) at a dilution of 1:300, anti-mouse F4/80 monoclonal antibody (clone BM8) #14-4801 (eBioscience, Hatfield, UK) at 1:100 dilution, and rabbit anti-MPO polyclonal antibody #1224 (Merck Millipore Corporation) at 1:1000 dilution. Visualization was with diaminobenzoate (DAB) according to standard protocols. Type-specific control antibodies were used to distinguish background staining. Immunohistochemistry slides were scanned in their entirety using an AxioScan.Z1 system (Zeiss microscopy GmbH, Oberkochen, Germany) and stored as.czi files before export as reduced-size.jpg files into ImageJ. Enumeration of caspase-3^+^, F4/80^+^, and MPO^+^ cells was done using ImageJ as previously described^12^, and expressed as positive cells per million pixels.

### RNA extraction and real-time PCR

Total RNA was extracted from kidney tissue using an RNeasy Mini kit (Qiagen). In all, 1 μg of total RNA was used for first-strand complementary DNA (cDNA) synthesis using a QuantiTect Reverse Transcription Kit (Qiagen). Expression of genes was determined by real-time PCR. Specific TaqMan primers and probes for *Kmo*, kynureninase (*Kynu*), *Kat2*, 3-hydroxyanthranilic acid oxidase (*Haao*), interleukin-6 (*Il6*), tumor necrosis factor α (*Tnfa*), chemokine (C-X-C motif) ligand 1 (*Cxcl1*), and ligand 2 (*Cxcl2*) were purchased from Life Biotechnologies. 18S ribosomal RNA was used as a reference gene. Amplification of cDNA samples was carried out using TaqMan® Fast Universal PCR Master Mix (AB Applied Biosystem) under the following conditions: 1-min denaturation at 95 °C, 45 cycles of 15 s at 95 °C, and 30 s at 60 °C. Thermal cycling and fluorescence detection were conducted in a StepOne real-time PCR system (Applied Biosystems). All reactions were carried out in triplicate and the cycle threshold (Ct) numbers of the target gene and reference gene in each sample were obtained. The mRNA levels of the target gene are presented as relative quantification (RQ) values.

### Liquid chromatography-tandem mass spectrometry (LC-MS/MS) analysis of kynurenine pathway metabolites

Samples of plasma were diluted at a ratio of 2:5 in 5 mM ammonium formate containing 0.1% trifluoracetic acid. Protein was precipitated by the addition of ice-cold 100% trichloroacetic acid to samples, followed by incubation for 30 min at 4 °C and centrifugation to obtain the supernatant. Serial dilutions of each metabolite were prepared over appropriate concentration ranges to prepare a calibration curve to permit quantitation. In all, 10 µL volumes of each sample were injected onto a Waters Select HSS XP column (30 mm × 100 mm, 2.5 µm, Waters Corp, Elstree, Herts) using a Waters Acquity UPLC autosampler, coupled to an ABSciex QTRAP 5500 mass analyzer. The flow rate was 0.35 mL/min at 25 °C. Separation was carried out using a water:methanol gradient (both containing 0.1% formic acid). Conditions were 50:50 water:methanol to 40:60 over 60 s, 40:60 to 35:65 over 180 s, hold 35:65 for 110 s, 35:65 to 50:50 over 10 s, and re-equilibration at 50:50 for 200 s. The total run time was 10 min. The mass spectrometer was operated in positive electrospray mode. The transitions for the protonated analytes were kynurenine, m/z 209-192; 3-hydroxykynurenine, m/z 225-202; tryptophan, m/z 205-188; kynurenic acid, m/z 190-144; and 3-hydroxyanthranilic acid, m/z 154-136. Collision energies were 29, 15, 11, 31, and 33 eV respectively. Data were acquired and processed using Analyst quantitation software (ABI Sciex).

### Tissue KMO enzyme activity assay

KMO enzyme activity assay in kidney tissue was determined as follows: 50–100 mg kidney tissue sample in 1 mL cold 0.32 μM sucrose solution was homogenized and then centrifuged at 1500 rpm for 5 min. Total protein concentration in the homogenate was determined using a Pierce BCA protein kit and adjusted to 2 mg/mL with 0.32 μM sucrose. In all, 10 μL of homogenate was added to 90 μL of master mix containing 200 μM kynurenine, 800 μM NADP, 3 mM G-6P, 1 unit of G6PD, 4 mM MgCI, and 2 mM HEPES with pH 7.4 in a well of a 96-well v-bottomed plate. The mix was incubated for 2 h at 37 °C with 250 rpm on an orbital shaker. In total, 500 μL of 100% acetonitrile, 250 ng/mL d5-tryptophan was added to each well after the incubation and the plate was centrifuged at 4000 rpm for 10 min. The supernatant was removed and dried under nitrogen at 65 °C, and then re-suspended in 100 μL of mobile phase (30:70 methanol:water containing 0.1% formic acids) for LC-MS analysis. Separation was carried out on a 5 μM, 100 × 4.6 mm Allure Biphenyl HPLC column using a methanol/water gradient at 50 °C with a flow rate of 500 μL/min. Typical retention times were 2.3 min for 3HK and 2.6 min for d5-tryptophan. The product ions of 3HK (tube lens = 75) and d5-tryptophan (internal standards, tube lens = 55) are detected. The peak area ratio (3HK area/d5-tryptophan area) for each sample is calculated and the amount of 3HK is determined from a standard curve of known 3HK concentrations (50, 100, 250, 500, 750, 1000, 1500, 2000 ng/mL). 3-Hydroxykynurenine was detected using LC-MS/MS.

### Experimental design and statistical analysis

A simple 2 × 2 factorial design was used to compare experimental IRI versus a sham procedure in *Kmo*^null^ and *Kmo*^wt^ mouse strains. Group sizes were determined by a prospective power calculation using G-power™^18^ using input parameters from previous IRI experiments using the same IRI method and background mouse strain. A detectable effect size of 0.80 with power 1-β = 0.80 and significance, *α* = 0.05, resulted in group sizes of *n* = 6 mice per group. All data are expressed as mean ± SEM. All data were subjected to a one-sample Kolmogorov–Smirnov test to check whether data adhered to a normal distribution. Normally distributed data were analyzed by one-way analysis of variance (ANOVA) followed by Tukey’s multiple comparison test. Data that did not follow a normal distribution were analyzed by Kruskal–Wallis test. All statistical analyses were performed using GraphPad™ Prism version 6.0d for Macintosh (GraphPad Software, San Diego, CA).

## Results

### Kmo^null^ mice are protected against AKI after renal IRI

**Fig. 2:**
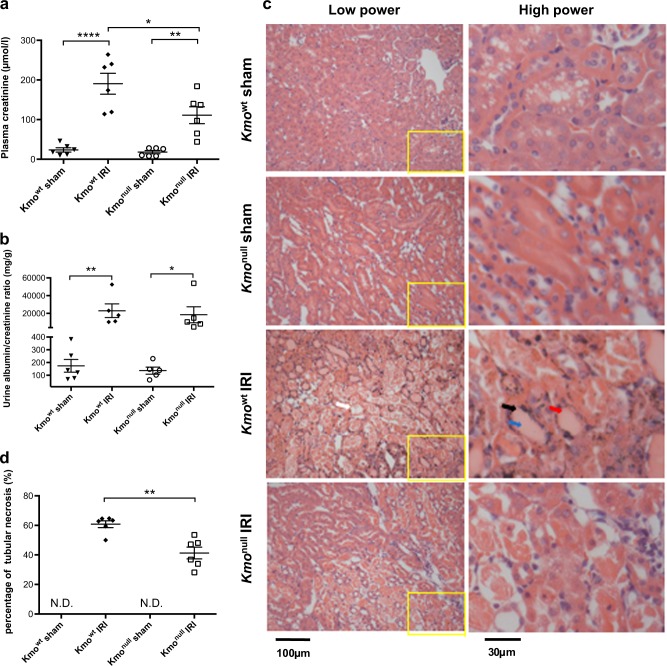
Biochemical indices of renal function and histological assessment of renal tubule injury after ischemia–reperfusion injury (IRI) in *Kmo*^wt^ and *Kmo*^null^ mice. **a** Plasma creatinine. **b** Urine albumin/creatinine ratio. **c** Representative digital micrographs of histological sections of formalin-fixed paraffin-embedded kidney tissue from *Kmo*^wt^ and *Kmo*^null^ mice after IRI or sham operation, stained with haematoxylin and eosin and visualized by light microscopy at × 200 original magnification. Low-power images are showed on the left panel. The relative image of selected area are showed in high power on the right panel. Tubular necrosis (white arrow), loss of the brush border (black arrow), cast formation (blue arrow), and tubular dilatation (red arrow) are indicated within the outer stripe of the renal medulla. **d** Enumeration of necrotic renal tubules expressed as a percentage of all tubules. N.D not detected. For all panels, mice were subjected to unilateral nephrectomy and contralateral IRI, or sham operation, under general anesthesia as described. Twenty-four hours after IRI or sham operation, mice were euthanased and blood, urine, and kidney sampled for analysis. All graphs show data from individual mice (one data point per mouse), with lines showing mean ± SEM. Group sizes were *n* = 6 mice per group, except for panel **b**, where urine was only successfully obtained from *n* = 5 mice per group (individual data shown). Statistically significant differences between groups were analyzed by one-way analysis of variance (ANOVA) with post hoc Tukey’s test; *P < 0.05, **P < 0.01 and ****P < 0.0001

To determine whether absent KMO activity affected the severity of AKI after IRI, we compared the effect of experimental IRI in mice. Mice on a C57BL/6 background engineered to lack KMO activity by insertion of a polyA transcription “stop” motif before exon 5 of the *Kmo* gene (*Kmo*^*tm1a(KOMP)Wtsi*^), hereafter referred to as *Kmo*^null^ mice, were generated and maintained in our laboratory as previously described^12^. Control mice (*Kmo*^*tm1c(KOMP)Wtsi/flox(ex5)*^), with normal *Kmo* gene transcription and KMO activity with *loxP*-flanked exon 5 of *Kmo* but no *cre*-recombinase expression and therefore a wild-type phenotype, hereafter referred to as *Kmo*^wt^ mice, were generated from the same founders as the *Kmo*^null^ strain. We have previously shown that KMO enzyme catalytic activity in these mice closely correlates with gene expression at mRNA level in both mouse strains and in multiple tissues^12^. *Kmo*^null^ mice were protected against the effects of renal IRI, experiencing less severe AKI, demonstrated by a lower plasma creatinine and less tubular damage than *Kmo*^wt^ control mice (Fig. 2). The experimental model of IRI was effective at inducing severe AKI, because IRI caused an elevated plasma creatinine to 190.5 ± 26.3 μmol/L at 24 h after IRI in *Kmo*^wt^ mice, compared with a plasma creatinine of 23.7 ± 5.5 μmol/L at the equivalent time after sham operation in *Kmo*^wt^ mice. *Kmo*^null^ mice had a lower plasma creatinine 24 h after IRI (111.2 ± 21.3 μmol/l) compared with the equivalent value in *Kmo*^wt^ control mice. This difference was statistically significant (*P* < 0.05) (Fig. 2a) with a magnitude that is biologically relevant. Renal IRI also significantly elevated the urinary ACR to 22,999 ± 7747 mg/g at 24 h after IRI in *Kmo*^wt^ mice, compared with an ACR of 174 ± 50 mg/g at the equivalent time after sham operation in *Kmo*^wt^ mice (*P* < 0.01, Fig. 2b and Supplementary Figures 1a and 1b). Urinary ACR was profoundly increased after IRI both in *Kmo*^wt^ and *Kmo*^null^, confirming AKI, but the magnitude of this elevation in *Kmo*^null^ is smaller than that in *Kmo*^wt^ mice although this difference is not statistically significant (*P* > 0.999, Fig. 2b). Because a previous report of a different strain of KMO-deficient mice^19^ reported proteinuria in unstressed KMO-deficient mice, we measured urine and plasma albumin concentrations (Supplementary Figure 1a and 1c). Urine albumin concentrations were not different between sham-operated *Kmo*^null^ and *Kmo*^wt^ mice, and not higher in *Kmo*^null^ mice.

When histological tubular damage was assessed, *Kmo*null mice were protected from structural AKI after IRI, congruent with the functional protection indicated by the plasma creatinine concentrations. *Kmo*^wt^ mice sustained severe tubular damage after IRI, evidenced by widespread tubular necrosis, loss of the brush border, cast formation, and tubular dilatation within the outer stripe of the outer medulla (Fig. 2c). On histological examination, kidneys from *Kmo*^null^ mice showed significantly less tubular damage after IRI compared with equivalent tissue sections from *Kmo*^wt^ mice. Kidneys from sham-operated mice from either *Kmo*^wt^ or *Kmo*^null^ strain incurred no visible tubular injury on histological assessment. Quantification of tissue injury, obtained by counting necrotic tubules expressed as a percentage of all tubules, was significantly lower in *Kmo*^null^ IRI mice (41.3 ± 4.0%) than *Kmo*^wt^ IRI mice (60.8 ± 2.3%) (*P* < 0.01, Fig. 2d). Together, these data clearly show that absent KMO activity in kidney tissue leads to a less severe functional and histological phenotype in AKI following experimental IRI.

### Tubular epithelial cell apoptosis is reduced in the kidney of Kmo^null^ mice following IRI

**Fig. 3:**
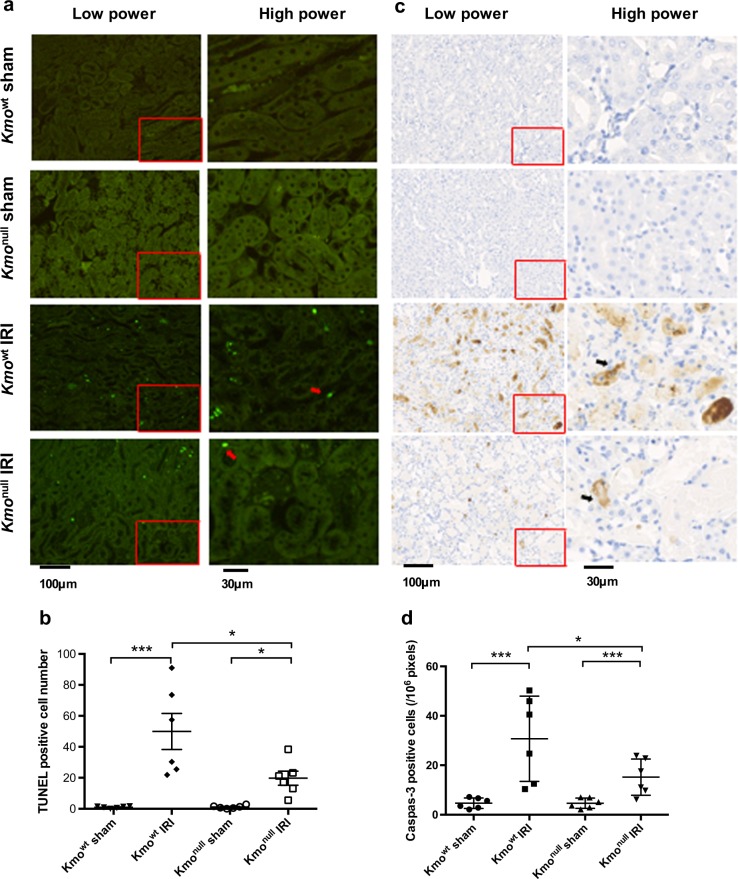
Detection of tubular epithelial cell apoptosis in kidney tissue after ischemia–reperfusion injury (IRI). **a** Representative digital micrographs of TdT-mediated dUTP nick-end labeling (TUNEL)-stained kidney sections at × 100 original magnification. Red arrows indicate TUNEL-positive apoptotic cells. Low-power images are showed on the left panel. The relative image of selected area are showed in high power on the right panel. **b** Number of TUNEL-positive cells per low-power field. **c** Representative digital micrographs selected from scanned caspase-3 stained entire kidney images. Black arrows indicate caspase-3-positive cells. Low-power images are showed on the left panel. The relative image of selected area are showed in high power on the right panel. **d** Cell apoptosis expressed as caspase-3-positive cells per 10^6^ pixels. For all panels, *Kmo*^wt^ and *Kmo*^null^ mice were subjected to IRI or sham operation as described and euthanased 24 h afterwards. Kidneys were sampled for analysis. Apoptotic cells were labeled by TUNEL assay and enumerated from digitally scanned micrographs using ImageJ. Apoptotic cells were also labeled by immunohistochemistry using antibodies to cleaved caspase-3, visualized by diaminobenzoate and enumerated. All graphs show data from individual mice (one data point per mouse), with lines showing mean ± SEM. Group sizes were *n* = 6 mice per group. Statistically significant differences between groups were analyzed by one-way analysis of variance (ANOVA) with post hoc Tukey’s test; *P < 0.05 and ***P < 0.001

Because therapeutic KMO inhibition protects against renal tubular cell apoptosis in AKI during experimental AP in rats, and because the KMO product 3-hydroxykynurenine induces apoptosis of cells in vitro, we examined the extent of tubular cell apoptosis. We labeled and enumerated apoptotic renal tubular cells using the TUNEL assay. There was no difference in the baseline apoptotic cell count in renal tubules in *Kmo*^wt^ and *Kmo*^null^ mice. Experimental IRI was a powerful inducer of apoptosis in renal tubular cells, causing substantial tubular epithelial cell apoptosis detectable at 24 h after IRI (Fig. 3a). Importantly, although *Kmo*^null^ mice sustained a degree of renal tubular cell apoptosis, the number of TUNEL-positive apoptotic renal tubular cells was significantly lower in *Kmo*^null^ mice with IRI (19.8 ± 4.5/field) than in *Kmo*^wt^ control mice (49.9 ± 11.6/field) (*P* < 0.05, Fig. 3b). Furthermore, immunohistochemistry using an antibody to cleaved caspase-3 corroborated the result obtained by TUNEL staining (Figs. 3c, d).

### KMO deletion inhibits neutrophil infiltration in the kidney following IRI

**Fig. 4:**
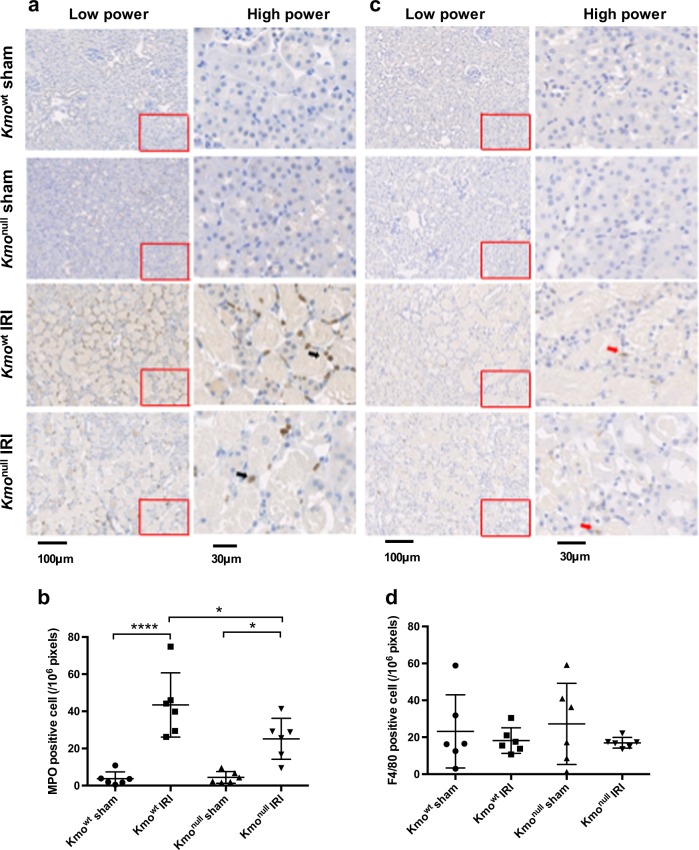
Neutrophil infiltration and monocyte-derived macrophage accumulation in kidney tissue after ischemia–reperfusion injury (IRI). **a** Representative digital micrographs selected from scanned myeloperoxidase (MPO)-stained entire kidney images. Black arrows indicate MPO^+^ cells. Low-power images are showed on the left panel. The relative image of selected area are showed in high power on the right panel. **b** MPO^+^ neutrophil infiltration, expressed as MPO^+^ cells per 10^6^ pixels. **c** Representative digital micrographs selected from scanned F4/80-stained entire kidney images. Red arrows indicate MPO^+^ cells. Low-power images are showed on the left panel. The relative image of selected area are showed in high power on the right panel. **d** F4/80^+^ monocyte-derived macrophage accumulation, expressed as F4/80^+^ cells per 10^6^ pixels. For all panels, *Kmo*^wt^ and *Kmo*^null^ mice were subjected to IRI or sham operation as described and euthanased 24 h afterwards. Kidneys were sampled for analysis. Neutrophils and monocyte-derived macrophages were labeled by immunohistochemistry using antibodies to myeloperoxidase (MPO) and F4/80, respectively, visualized by diaminobenzoate and enumerated. All graphs show data from individual mice (one data point per mouse), with lines showing mean ± SEM. Group sizes were *n* = 6 mice per group. Statistically significant differences between groups were analyzed by one-way analysis of variance (ANOVA) with post hoc Tukey’s test; *P < 0.05 and ****P < 0.0001

AKI incorporates an element of acute inflammation. Therefore, we directly measured neutrophil accumulation in the kidney following IRI by immunohistochemical staining for MPO and enumeration of MPO^+^ cells. A clearly measurable influx of MPO^+^ neutrophils was identified on sections of kidney tissue in both *Kmo*^wt^ and *Kmo*^null^ mice, 24 h after IRI, compared with very low numbers of MPO^+^ cells in kidneys of sham-operated animals (Fig. 4a). Using quantitative analysis, we detected a lower number of accumulated neutrophils in the kidneys of *Kmo*^null^ mice (25.2 ± 4.5/10^6^ pixels) compared with *Kmo*^wt^ mice after IRI (43.4 ± 7.1/10^6^ pixels), and this difference was statistically significant (*P* < 0.05, Fig. 4b). We also examined monocyte-derived macrophage accumulation after IRI by F4/80 immunohistochemistry (Fig. 4c). There was no significant infiltrate of cells positive for the mouse monocyte marker F4/80 in the kidney at 24 h after renal IRI in either *Kmo*^null^ mice (17.0 ± 1.2/10^6^ pixels) or *Kmo*^wt^ mice (18.2 ± 2.8/10^6^ pixels) (Fig. 4d).

### Renal IRI downregulates kynurenine pathway enzyme mRNA expression and upregulates interleukin-6 and tumor necrosis factor-α mRNA expression

**Fig. 5:**
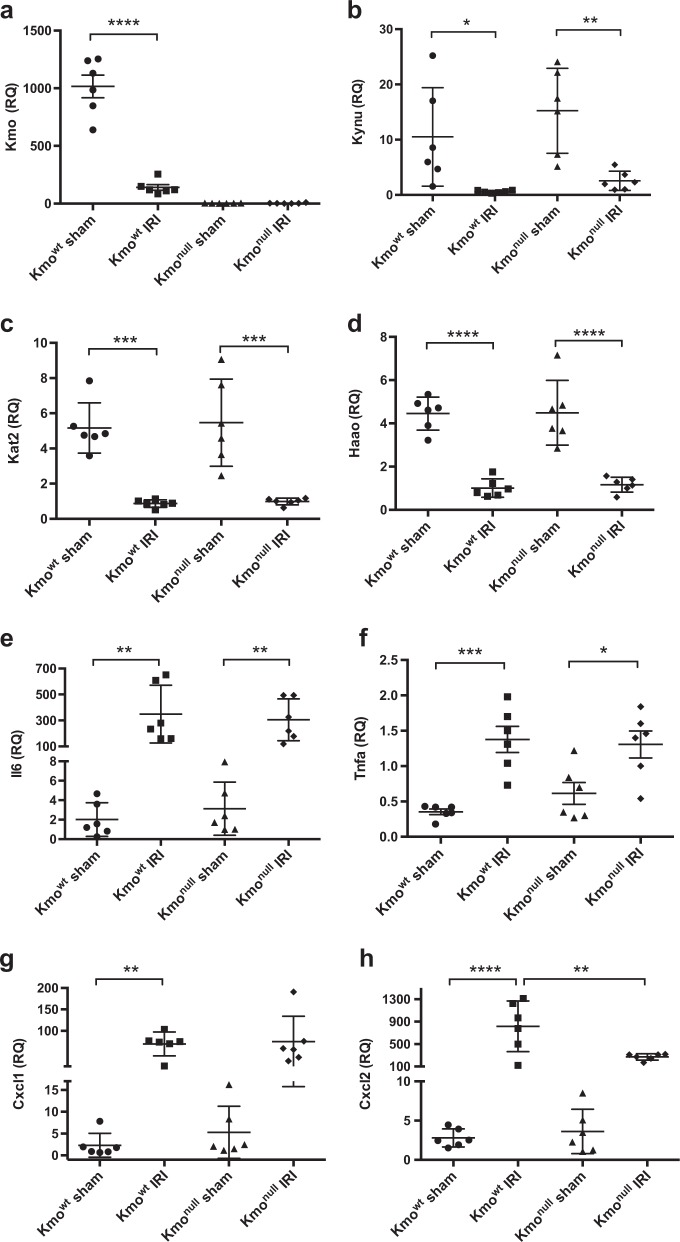
mRNA expression of kynurenine pathway enzymes and pro-inflammatory cytokines in kidney tissue after ischemia–reperfusion injury (IRI). **a** Kynurenine 3-monoxygenase, *Kmo*; **b** kynureninase, *Kynu*; **c** kynurenine aminotransferase, *Kat2*; **d** 3-hydroxyanthranilic acid oxidase, *Haao*; **e** interleukin-6, *Il6*; **f** tumor necrosis factor α, *Tnfa*. **g** chemokine (C-X-C motif) ligand 1, *Cxcl1*; **h** chemokine (C-X-C motif) ligand 2, *Cxcl2*. For all panels, *Kmo*^wt^ and *Kmo*^null^ mice were subjected to IRI or sham operation and euthanased 24 h afterwards. Kidney tissue was sampled, snap frozen in liquid nitrogen, and RNA subsequently extracted for analysis by real-time PCR as described. mRNA levels of the target gene were normalized to 18S ribosomal RNA and are presented as relative quantification (RQ) values. All graphs show data from individual mice with lines showing mean ± SEM. Group sizes were *n* = 6 mice per group. Statistically significant differences between groups were analyzed by one-way analysis of variance (ANOVA) with post hoc Tukey’s test; *P < 0.05, **P < 0.01, ***P < 0.001 and ****P < 0.0001

Next, we asked whether IRI affected expression of kynurenine pathway enzymes, specifically *Kmo*, kynureninase (*Kynu*), *Kat2*, 3-hydroxyanthranilic acid oxygenase (*Haao*), and Quinolinate phosphoribosyltransferase (*Qprt)* in kidney tissue. Using real-time PCR, we detected a profound and statistically significant decrease in *Kmo* mRNA expression in kidney tissue after IRI in *Kmo*^wt^ mice compared with baseline expression levels in sham-operated *Kmo*^wt^ mice. There was no detectable *Kmo* mRNA expression in kidneys from *Kmo*^null^ mice, in keeping with the expected genotype and with our previous experience with this mouse strain (Fig. 5a). Interestingly, IRI caused a reduction in mRNA expression in both *Kmo*^wt^ and *Kmo*^null^ mouse strains for *Kynu, Kat2*, *Haao*, and *Qprt* when compared with sham-operated mice of both transgenic strains. There was no strain-specific difference in the expression of *Kynu, Kat2*, *Haao*, and *Qprt* mRNA attributable to *Kmo* gene deletion in *Kmo*^null^ mice (Figs. 5b, c, d and supplementary Figure 2). The observed reduction in kynurenine pathway enzyme mRNA expression cannot be explained by experimental artifact, because quantification of mRNA expression of the pro-inflammatory cytokines interleukin-6 (IL-6) and tumor necrosis factor-α (TNFα) in the same cDNA preparations used for kynurenine pathway transcripts was significantly upregulated following IRI, and equally so in *Kmo*^wt^ and *Kmo*^null^ mouse strains. Sham-operated mouse kidneys from both *Kmo*^wt^ and *Kmo*^null^ strains had very low expression of IL-6 and TNFα mRNA. Mice subjected to IRI demonstrated strong upregulation of IL-6 and TNFα compared with sham-operated mice: IL-6 mRNA expression in the IRI kidney increased 173-fold in *Kmo*^wt^ mice and 97.3-fold in *Kmo*^null^ mice, respectively (Fig. 5e); TNFα mRNA expression in the IRI kidney increased 3.9-fold in *Kmo*^wt^ mice and 2.1-fold in *Kmo*^null^ mice (Fig. 5f). The difference in IL-6 and TNFα mRNA upregulation in IRI was not statistically significant between *Kmo*^wt^ mice and *Kmo*^null^ mice. We also measured chemokine (C-X-C motif) ligand 1 (*Cxcl1*) and ligand 2 (*Cxcl2*) mRNA levels in kidney tissue because these regulate the recruitment and infiltration of polymorphonuclear cells (PMN). Cxcl1 and Cxcl2 mRNA expression in IRI kidney tissue was significantly increased in *Kmo*^wt^ mice. Cxcl2 mRNA level in *Kmo*^null^ mice was lower compared with *Kmo*^wt^ mice after IRI; this difference was statistically significant (*P* < 0.001), and congruent with the observation of a reduced neutrophil infiltrate. However, there was no difference between Cxcl1 mRNA levels in IRI kidney tissue from *Kmo*^wt^ mice and *Kmo*^null^ mice (Figs. 5g, h**)**. Together, these data show that renal IRI drives a downregulation of kynurenine pathway enzyme mRNA expression, or potentially reflects loss of tubular cells through necrosis, independent of the functionality of *Kmo*, and simultaneously upregulates pro-inflammatory cytokine expression.

### Kynurenine metabolite changes in experimental IRI in mice

**Fig. 6:**
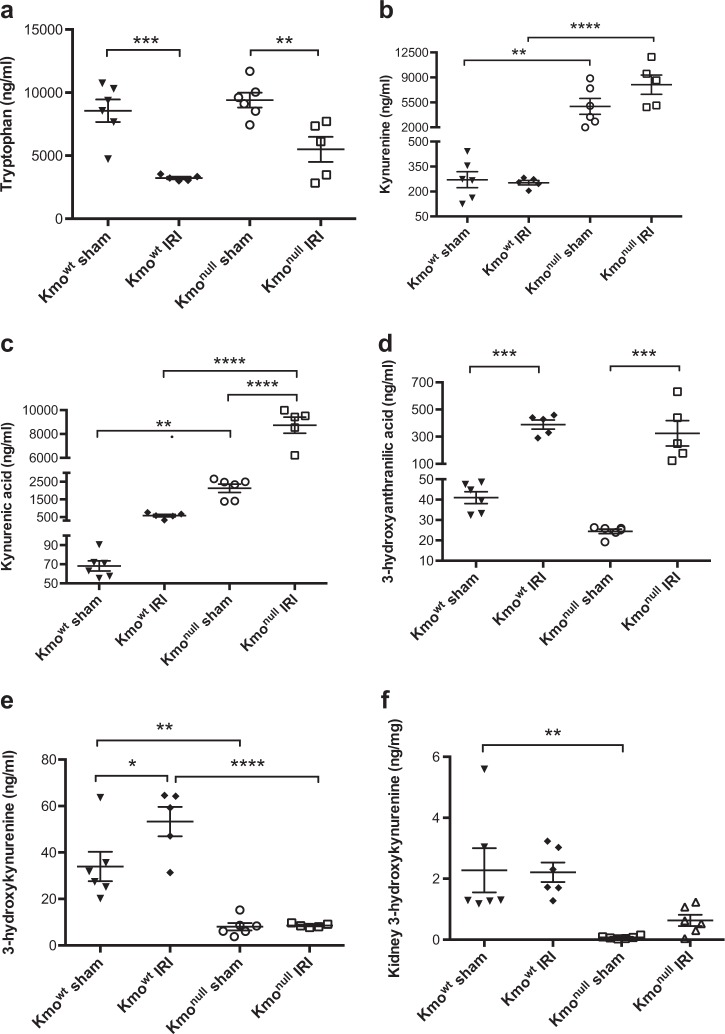
Kynurenine pathway metabolite concentrations in plasma and kidney tissue after ischemia–reperfusion injury (IRI). **a** Plasma tryptophan; **b** plasma kynurenine; **c** plasma kynurenic acid; **d** plasma 3-hydroxyanthranilic acid; **e** plasma 3-hydroxykynurenine; **f** kidney tissue 3-hydroxykynurenine. For all panels, mice were subjected to unilateral nephrectomy and contralateral IRI, or sham operation, under general anesthesia. Twenty-four hours after IRI or sham operation, mice were euthanased and blood and kidney tissue sampled for analysis. Extracts of plasma (panels **a–e**) or kynurenine 3-monooxygenase (KMO) activity in kidney tissue (panel **f**) were analyzed by liquid chromatography-tandem mass spectrometry (LC-MS/MS) as described. All graphs show data from individual mice (one data point per mouse), with lines showing mean ± SEM. Group sizes were *n* = 6 or *n* = 5 (where one plasma was not obtained) mice per group. Statistically significant differences between groups were analyzed by one-way analysis of variance (ANOVA) with post hoc Tukey’s test; *P < 0.05, **P < 0.01, ***P < 0.001 and ****P < 0.0001

The metabolic product of KMO, 3-hydroxykynurenine, is injurious to cells and tissues. Because *Kmo*^null^ mice are unable to form 3-hydroxykynurenine and are also protected from AKI, it was important to confirm absent 3-hydroxykynurenine in plasma and kidney tissue after IRI. Furthermore, because the biochemical phenotype of *Kmo*^null^ mice is a diversion of kynurenine metabolism to kynurenic acid, and because we had observed that IRI downregulates kynurenine pathway enzyme mRNA expression, we measured kynurenine metabolite concentrations in plasma and tissue (Fig. 6). In sham-operated animals, we observed the plasma biochemical phenotype expected of *Kmo*^null^ mice, namely reduced (or absent) 3-hydroxykynurenine levels, reduced 3-hydroxyanthranilic acid levels, an upstream backlog of kynurenine, and a metabolic diversion of kynurenine to kynurenic acid. Tryptophan concentrations were not different between sham-operated *Kmo*^wt^ and *Kmo*^null^ mice. After IRI, the kynurenine pathway biochemical phenotype showed intriguing changes: plasma tryptophan was depleted. Tryptophan concentration in *Kmo*^null^ mice was significantly lower in the IRI group (5501 ± 999 ng/mL) than in sham-operated group (9411.0 ± 588.2 ng/mL) (*P* < 0.01). Tryptophan concentration in *Kmo*^wt^ mice was also significantly lower in the IRI group (3232 ± 98.6 ng/mL) than in sham-operated group (8560 ± 893.5 ng/mL) (*P* < 0.001). The degree of tryptophan depletion was less pronounced in *Kmo*^null^ mice than *Kmo*^wt^ mice after IRI, but this was not statistically significant between these two mouse strains (Fig. 6a). Kynurenine concentrations were not altered in IRI to an extent that exceeded the profound differences at baseline between *Kmo*^wt^ and *Kmo*^null^ mouse strains (Fig. 6b). Experimental IRI caused an elevation in the potentially protective molecule kynurenic acid that was significantly further increased in *Kmo*^null^ mice with IRI (*Kmo*^null^ IRI 8738.0 ± 673.6 ng/mL and *Kmo*^wt^ IRI 581.6 ± 72.9 ng/mL; *P* < 0.0001, Fig. 6c). Although IRI induced a rise in 3-hydroxyanthranilic acid, this was not different between *Kmo*^wt^ and *Kmo*^null^ mouse strains (*Kmo*^null^ IRI 324.3 ± 93.6 ng/mL and *Kmo*^wt^ IRI 389.0 ± 33.3 ng/mL; Figure 6d). Concentrations of 3-hydroxykynurenine were significantly increased 24 h after IRI in *Kmo*^wt^ mice in plasma (IRI 53.3 ± 6.3 ng/mL and sham 34.0 ± 6.3 ng/mL; *P* < 0.05, Fig. 6e), but not in kidney tissue (Fig. 6f), and, as expected, *Kmo*^null^ mice showed extremely low levels of 3-hydroxykynurenine in plasma and KMO activity in kidney tissue (Figs. 6e, f). Together, these data convincingly demonstrate that 3-hydroxykynurenine production by KMO is a significant contributor to AKI following renal IRI, and reinforce the concept of KMO inhibition as a protective strategy to protect against organ dysfunction in critical illness.

## Discussion

Kynurenine metabolites are generated by tryptophan catabolism and regulate biological processes that include host–microbiome signaling, immune cell response, and neuronal excitability^20^. More recently, we and others are focusing on the role of the kynurenine pathway in inflammation and tissue injury. In particular, we have focused on the pathway enzyme KMO, which plays a key gate-keeper role in kynurenine metabolism, by determining the metabolic fate of kynurenine generated by upstream dioxygenases. Recent genetic analysis in mice identified Kmo as a candidate gene associated with albuminuria. Glomerular KMO expression is decreased in both mouse and human kidneys in a diabetic environment^19^. These previous reports support a link between KMO expression and healthy kidney function. In the present work, we conclusively demonstrate that KMO is a critical regulator of tissue injury in kidneys subjected to IRI. We used a transgenic mouse strain (*Kmo*^null^ mice) that lacks KMO activity in all tissues by disruption of transcription in exon 5 of the *Kmo* gene, and compared it with *Kmo*^wt^ mice with normal KMO activity. The key finding of our paper is that *Kmo*^null^ mice are significantly protected from AKI after IRI, measured by preserved biochemical renal function and histological evidence of protection against tubule cell necrosis and apoptosis. Furthermore, neutrophil infiltration was reduced in *Kmo*^null^ mice with IRI, when compared with *Kmo*^wt^ mice as controls.

Tryptophan metabolism through the kynurenine pathway is most widely studied to date in relation to disorders of the nervous system, for example, Huntington’s disease, stress-related depression, schizophrenia, Alzheimer’s disease, and Parkinson’s disease^21^. In recent years, the regulation of kynurenine metabolism has been increasingly evaluated in relation to acute tissue injury, and has become an attractive therapeutic target in several disease areas^22^. Our data here show altered kynurenine metabolite levels in *Kmo*^null^ mice after IRI compared with *Kmo*^wt^ IRI mice. Despite showing comparable downregulated expression of the kynurenine pathway enzymes following experimental IRI, levels of kynurenine and kynurenic acid were significantly higher in *Kmo*^null^ mice compared with *Kmo*^wt^ controls. Although such changes are characteristic of the *Kmo*^null^ phenotype, kynurenine and kynurenic acid levels were further increased in *Kmo*^null^ IRI mice compared with *Kmo*^null^ sham-operated mice. These metabolites may contribute to protection from AKI after IRI in *Kmo*^null^ mice, particularly since kynurenic acid has demonstrated a protective role in a number of other inflammatory situations through its activity at glutamate receptors. Levels of free radical-generating 3-hydroxyanthranalic acid were increased to a similar degree in both mouse strains following induction of IRI suggesting that this metabolite is not implicated in the development of AKI. As plasma 3HK production was significantly higher in *Kmo*^wt^ IRI mice than in all other conditions, it seems likely that 3HK-mediated effects contribute to the observed exacerbated response to IRI. This study provides further evidence that metabolic flux through the kynurenine pathway is upregulated in systemic inflammation, congruent with clinical observations in humans with AP^23,24^, trauma^25,26^, undergoing coronary artery bypass surgery^27^, sepsis^28^, and chronic renal failure^29^. Plasma concentrations of 3-hydroxykynurenine correlate with the burden of inflammation, incidence of organ dysfunction and AP severity in human^24^. Altered kynurenine pathway metabolite levels observed here suggest that KMO also plays a critical role in kidney injury, strengthening the rationale for the use of systemic KMO inhibitors in this indication.

*Kmo*^null^ mice were generated and characterized in our laboratory to investigate the role of 3-hyxdroxykynurenine as an important effector of tissue injury. In *Kmo*^null^ mice, fecundity, fertility, and longevity up to 2 years of age are not affected, from which we can infer that prolonged KMO blockade and consequent chronic exposure to significantly elevated concentrations of kynurenic acid and kynurenine is well tolerated, at least in adapted mice^12^. Other researchers have also independently demonstrated that the deleted *Kmo* was not essential for embryonic and postnatal survival^30^. Our finding that *Kmo*^null^ mice do not have albuminuria at baseline is contrary to a report from others^19^, but could possibly explained by difference in C57BL/6 substrain. The background strain used by Konstanje et al.^19^ was C57BL/6N and the background strain of our mice used in this article is C57BL/6J. Interestingly, deleting of arylformamidase (Afmid) in mice leads to a glomerulosclerosis phenotype. However, the *Afmid* mouse also has a thymidine kinase promoter deleted and a glucose intolerance phenotype with reduced insulin secretion and therefore is not a clean model from which a direct inference of an effect on kynurenine metabolism on renal function can be drawn^31,32^. KMO depletion decreased plasma 3HK level and increased plasma KA level, which may be potentially protective against renal damage caused by IRI.

Kidney IRI is an inevitable consequence of the procedure of kidney transplantation and its severity has been correlated with an increased incidence of both acute and chronic rejection^17,33^. Apoptosis of tubular epithelial cells contributes to the development of ischemic AKI, and injury at this site contributes to organ failure. Inhibiting apoptosis, both before and after renal insults, will inevitably improve the outcome of human AKI^34^. Neutrophils and monocytes/macrophages are important contributors to ischemic kidney injury and repair. Neutrophils attach to the activated endothelium and accumulate in the kidney both in animal models and in human AKI^35^. Recent studies have shown that pro-inflammatory cytokines and chemokines such as TNF-α, IL-6, monocyte chemoattractant protein 1, and MIP-2 contribute to the development of renal IRI^36^. In the present study, we report the original finding that genetic KMO deletion provides protection against kidney damage caused by IRI. Renal injury was observed on histological sections of kidney tissue from mice with IRI. They also showed an increase in neutrophilic inflammation and increased apoptosis. All these features of renal injury were essentially reduced in KMO depletion mice. Additionally, we observed that the mRNA expression of pro-inflammatory cytokines such as TNF-α and IL-6 increased in IRI mice compared with sham animals, which has been reported earlier in human AP patients^24^. There was no significant difference about the TNFα and IL-6 mRNA expression between *Kmo*^null^ and *Kmo*^wt^ IRI mice. The chemokines C-X-C motif ligand 1 (CXCL1)/keratinocyte chemoattractant (KC), C-X-C motif ligand 2 (CXCL2)/macrophage inflammatory protein-2 (MIP-2) have been found to contribute to the pathology of a number of neutrophil-dependent animal models of disease, including IRI^37–39^. Our study showed that renal CXCL1 and CXCL2 mRNA expression were upregulated in IRI *Kmo*^wt^ mice compared with sham-operated control mice. Lower CXCL2 mRNA levels in *Kmo*^null^ IRI mice compared with *Kmo*^wt^ IRI mice are consistent with our observation of reduced neutrophil infiltration.

In summary, our study shows *Kmo*^null^ mice had preserved renal function, reduced renal tubule cell injury and apoptosis, and fewer infiltrating neutrophils compared with *Kmo*^wt^ control mice. Together, these data strongly support the translational potential of KMO inhibition as a therapeutic strategy to protect against renal injury in acute inflammation.

## Supplementary information

Supplementary Material
