# Supplementary material for: Kynurenine 3-monooxygenase is a critical regulator of renal ischemia–reperfusion injury
Source: Exp Mol Med. 2019 Feb 13;51(2):15. doi: 10.1038/s12276-019-0210-x (PMC6374422; doi:10.1038/s12276-019-0210-x)
Supplement: Supplementary file 1 — Supplementary Material [file 12276_2019_210_MOESM1_ESM.pdf]

Kynurenine 3-monooxygenase (KMO) is a critical regulator of renal ischemia-reperfusion injury. Xiaozhong Zheng[1], Ailiang Zhang[1], Margaret Binnie[2], Kris McGuire[2], Scott P Webster[2], Jeremy Hughes[1], Sarah E M Howie[1] and Damian J Mole[1,3,*]

SUPPLEMENTAL INFORMATION

Supplementary Figure 1

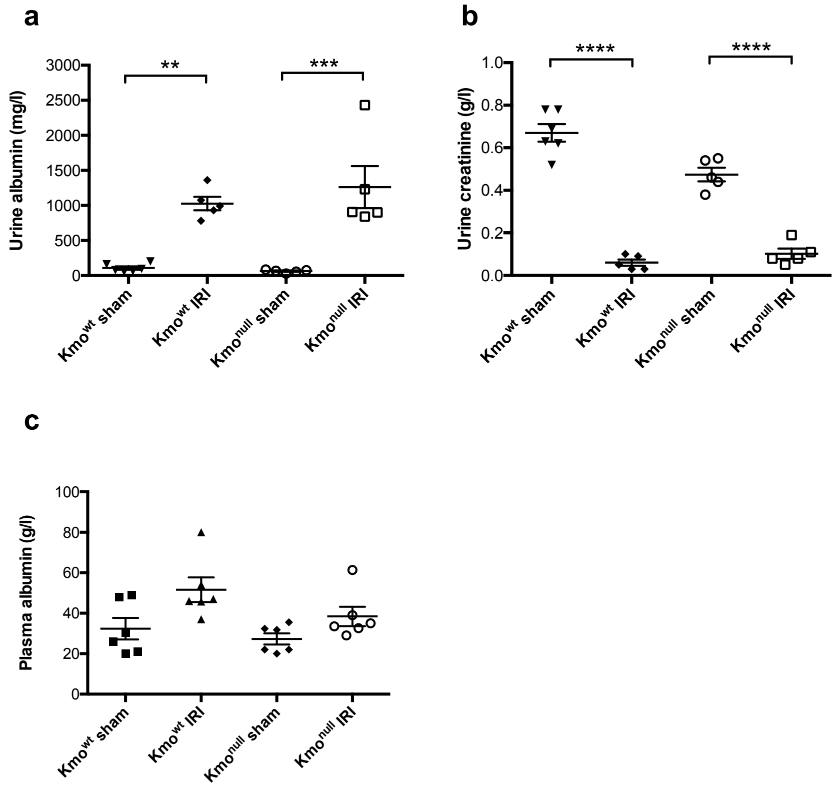

Supplementary figure legends

Supplementary figure 1. Urine albumin and creatinine and plasma albumin in *Kmo*^{wt} and *Kmo*^{null} mice after ischemia-reperfusion injury (IRI). (**a**) urine albumin; (**b**) urine creatinine; (**c**) Plasma albumin. The graphs show data from individual mice (one data point per mouse), with lines showing mean ± SEM. Group sizes were n=6 mice per group, except for panel **a** and **b**, where urine was only successfully obtained from n=5 mice for some groups (individual data shown). Statistically-significant differences between groups were analysed by one-way ANOVA with post hoc Tukey's test; ** P<0.01; *** P<0.001 and **** P<0.0001.

Supplementary Figure 2

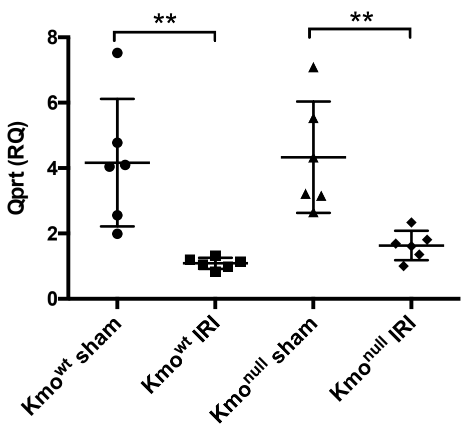

Supplementary figure 2. Quinolinate phosphoribosyltransferase (*Qprt)* mRNA expression in kidney tissue of *Kmo*^{wt} and *Kmo*^{null} mice after IRI. mRNA levels of the target gene were normalized to 18S ribosomal RNA and are presented as relative quantification (RQ) values. The graph shows data from individual mice with lines showing mean ± SEM. Group sizes were n=6 mice per group. Statistically-significant differences between groups were analysed by one-way ANOVA with post hoc Tukey's test; ** P<0.01.
